# GPR30-mediated non-classic estrogen pathway in mast cells participates in endometriosis pain *via* the production of FGF2

**DOI:** 10.3389/fimmu.2023.1106771

**Published:** 2023-02-08

**Authors:** Xinxin Xu, Jianzhang Wang, Xinyue Guo, Yichen Chen, Shaojie Ding, Gen Zou, Libo Zhu, Tiantian Li, Xinmei Zhang

**Affiliations:** ^1^ Department of Gynecology, Women’s Hospital, Zhejiang University School of Medicine, Hangzhou, Zhejiang, China; ^2^ Zhejiang Province Key Laboratory of Precision Diagnosis and Therapy for Major Gynecological Diseases, Women’s Hospital, Zhejiang University School of Medicine, Hangzhou, Zhejiang, China; ^3^ Department of Gyneclogy, Ningbo Women and Children’s Hospital, Ningbo, Zhejiang, China

**Keywords:** endometriosis, mast cell, G-protein coupled receptor 30, fibroblast growth factor 2, pain

## Abstract

Pain is one of the main clinical symptoms of endometriosis, but its underlying mechanism is still not clear. Recent studies have shown that the secretory mediators of mast cells activated by estrogen are involved in the pathogenesis of endometriosis-related pain, but how estrogen-induced mast cell mediators are involved in endometriosis-related pain remains unclear. Here, mast cells were found to be increased in the ovarian endometriotic lesions of patients. They were also closely located closely to the nerve fibers in the ovarian endometriotic lesions from of patients with pain symptoms. Moreover, fibroblast growth factor 2 (FGF2)-positive mast cells were upregulated in endometriotic lesions. The concentration of FGF2 in ascites and the protein level of fibroblast growth factor receptor 1 (FGFR1) were higher in patients with endometriosis than in those without endometriosis, and they were correlated with pain symptoms. *In vitro*, estrogen could promote the secretion of FGF2 through G-protein-coupled estrogen receptor 30 (GPR30) *via* the MEK/ERK pathway in rodent mast cells. Estrogen-stimulated mast cells enhanced the concentration of FGF2 in endometriotic lesions and aggravated endometriosis-related pain *in vivo*. Targeted inhibition of the FGF2 receptor significantly restrained the neurite outgrowth and calcium influx in dorsal root ganglion (DRG) cells. Administration of FGFR1 inhibitor remarkably elevated the mechanical pain threshold (MPT) and prolonged the heat source latency (HSL) in a rat model of endometriosis. These results suggested that the up-regulated production of FGF2 by mast cells through non-classic estrogen receptor GPR30 plays a vital role in the pathogenesis of endometriosis-related pain.

## Introduction

Endometriosis is an estrogen-dependent chronic inflammatory disorder affecting 10% of reproductive-age women worldwide (1, 2). Women with endometriosis experience debilitating pelvic pain, such as dysmenorrhea, dyspareunia, dyschezia, and chronic pelvic pain (3, 4). Options for managing endometriosis-related pain have long been limited because the pathogenesis of endometriosis-related pain remains ambiguous (5).

Current perspectives considered endometriosis-related pain as an inflammatory pain (6). Nerve fibers distributed in endometriotic lesions (6, 7) could be activated by the local neuroinflammatory environment (8, 9). The interaction between nociceptors and cytokines has been shown to trigger nerve sensitization, leading to hyperalgesia and causing chronic pain (10–12). Thus, exploring the imbalanced state of various immune inflammatory cells infiltrating around endometriotic lesions is important in exploring potential directions for pain management in endometriosis. Mast cells are granular immune cells settled in tissues. Various receptors and mediators of mast cells are important for in linking the immune response and playing an important role in many inflammatory diseases, tissue remodeling, and antitumor immune responses (13–16). The function of mast cells in interacting with sensory nerve cells has been widely reported in interstitial cystitis and irritable bowel syndrome (17, 18). To date, limited attention has been paid to the role of mast cells in endometriosis-related pain (19), although researchers found the number and distance of mast cells located around nerve structures were associated with the pain and itch (20, 21).

Some studies have found that ERα and ERβ were located in mast cells in the human reproductive system which indicating that estrogen may regulate the function of mast cells in the local environment and play a role in pain (22, 23). Our previous study proved the increasing mast cells in the endometriotic lesions by immunohistochemistry and revealed the vital role of estrogen in mediating mast cell activation in endometriosis-associated dysmenorrhea by experiments, such as estrogen concentration measurement, mast cell degranulation quantitation, and cell migration assay (9). Estrogen plays a role by binding its receptors, including nuclear estrogen receptors (ERα and ERβ) and the membrane estrogen receptor (GPR30) (24). Our recent research confirmed that estrogen could regulate estrogen response element (ERE) through Erα, thus modulating the function of mast cells in endometriotic lesions (25). However, whether estrogen affects the function of mast cells *via* G-protein coupled estrogen receptor 30 (GPR30) remains unclear. A previous study found that GPR30 was able to mediate the effect of estrogen in mast cells (26). Other researchers proved that visceral hypersensitivity in stressed rats were mediated by estrogen *via* GPR30 (27). However, the relationship between GPR30-mediated estrogen pathway in mast cells and endometriosis-related pain attracted little concern, and its underlying mechanisms need to be investigated further.

The interactions between mast cells and sensory nerves have been found in many chronic inflammatory diseases, such as psoriasis, contact dermatitis, celiac disease, and endometriosis (19, 28–32). Mast cells may activate the primary sensory nerve fibers close to them by releasing their own media, resulting in the release of glutamate and neurotransmitters, the activation of voltage-gated calcium channels, and the activation of microglial neurons in the spinal cord, thus causing nerve sensitization and related pain symptoms (33, 34). According to literature, the research on mast cell-derived fibroblast growth factor (FGF2) mainly focuses on angiogenesis, fibrosis, wound healing, hypertension, kidney damage, airway hyperresponsiveness, chronic obstructive pulmonary disease, and other diseases (35–38). Although some studies have involved FGF2 and endometriosis, the source of elevated FGF2 in the lesion (39, 40), peritoneal fluid (41), or peripheral blood (42, 43) and its role in the pain associated with endometriosis need to be further explored. Many studies have used dorsal root ganglion (DRG) cells to study the effect of nerve growth factor, thromboxane A2 derived from peritoneal fluid of patients with endometriosis, and insulin-like growth factor 1 derived from macrophages on nerve growth, thus proving its involvement in the generation of pain symptoms in endometriosis (12, 44, 45).

This study proved that estrogen induced mast cell activation through non-classical estrogen receptors *via* the MEK/ERK pathway in endometriosis. The upregulated FGF2 by activated mast cells could promote the neurite outgrowth and calcium influx of DRG cells. In addition, introducing the fibroblast growth factor receptor 1 (FGFR1) inhibitor NSC12 could reverse the effect of FGF2 in DRG cells and reduce the pain hyperalgesia of endometriosis rats.

## Materials and methods

### Patient enrollment and sample collection

This study was approved by the Human Ethics Committee of Women’s Hospital, School of Medicine, Zhejiang University (No.20160114). All patient signed an informed consent to participate in this study, and they were able to opt out of the study for the whole duration. A total of 32 ovarian endometriotic lesions from women with endometriosis and 16 endometrium tissues from patients without endometriosis were collected and immersed in liquid nitrogen for RNA or protein extraction. Among them, 16 ovarian endometriotic lesions from women with endometriosis and 11 endometrium tissues from patients without endometriosis were fixed in formalin as well for histology examination. Peritoneal fluid samples from 53 patients with endometriosis (including peritoneal endometriosis, PE; ovarian endometriosis, OE; deep infiltrated endometriosis, DIE) and 16 patients without endometriosis were collected during the surgery, transported at 4°C, and stored at –80°C after centrifuging. The patients without endometriosis were those who underwent surgical procedures for myoma, ovarian teratoma, and hydrosalpinx. The severity of pain was assessed using a Visual Analog Scale (VAS). Self- reported endometriosis-associated pain was measured by VAS on a scale with a range from 0 to 10. A score of 1–3 was considered mild, 4–6 as moderate and >6 as severe pain. None of the patients received sex-hormone therapy 6 months before surgery. (Clinical information of patients were listed in the **Additional file 1**).

### Cell culture

Rat mast cell line: RBL2H3 cells are rat basophilic leukemia cells obtained from the Cell Bank of the Chinese Academy of Sciences (#SCSP-518; Shanghai, China). They were cultured in MEM medium (Gibco) containing 15% fetal bovine serum (FBS; Sigma Aldrich). DRG cell line: The F11 cells were obtained from the European Collection of Authenticated Cell Cultures (# 08062601; Salisbury, UK). The F11 cells are a somatic cell hybrid of a rat embryonic DRG and mouse neuroblastoma cell line N18TG2. The line retains both rat and mouse chromosomes and synthesizes both rat and mouse isoenzymes. They were maintained in DMEM/F12 medium (Gibco) supplemented with 10% FBS. All these cell lines were cultured at 37°C and 5% CO2, with a change of the medium every 2–3 days.

### Generation of GPR30 knockout cell line with CRISPR/Cas9

The guide RNA sequences for CRISPR/Cas9 were designed using the online design tool: CRISPOR (http://crispor.tefor.net/). The common CDS region of all transcripts of *GPER1* (Gene ID:171104) was selected, and the first exon where the common CDS region is located was found for target site design. Oligonucleotides were listed as follows: gRNA1: 5′ – ACTACTCCAGCACAAGATGTTGG - 3′; gRNA2: 5′– A GATCTACCTAGGTCCCGTGTGG- 3′; gRNA3: 5′ – GGCGCTGCGGGAAGATG CCCCGG-3′. RBL2H3 cells were transfected with pGK1.1/gRNA using electroporation (Cat. No. GP7910, Genloci Biotechnologies Inc. China). Seventy-two hours after transfection, the cells were treated with 1 μg/mL of puromycin for 3 days. After 2 weeks, colonies were isolated with the cloning cylinders, and the cells were analyzed with sequencing.

### Different interventions of cells

RBL2H3 and *Gper1* KO cells were treated with various concentrations of 17β-estradiol (1 nmol/L, 10 nmol/L, 100 nmol/L, 1 μmol/L,10 μmol/L) or serum-free medium for 24 h. The supernatant and cells of each group were collected and stored at –80°C for further experiment. The supernatant from RBL2H3 and *Gper1* KO cells with or without 100 nmol/L 17β-estradiol were added into F11 cells. Twenty-four hours later, the neurite outgrowth of F11 cells were detected among the groups. The calcium influx of F11 cells were detected among the groups after the supernatant adding. For signal pathway research, RBL2H3 and *Gper1* KO cells were treated with 100 nmol/L 17β-estradiol at different timepoints (5 min, 15 min, 30 min, 1h, 3h, 6h, 12 h, 24 h), the cells of each group were collected for protein extraction. In addition, RBL2H3 and *Gper1* KO cells were preincubated with or without 10 μmol/L PD98059 for 1 h, and treated with 100 nmol/L 17β-estradiol for 3 h, the cells of each group were collected for protein extraction. For the part of the NSC12 intervention, the F11 cells were treated with 200 ng/mL FGF2 for 24 h with or without NSC12 (10 μmol/L, preincubated for 12 h). Serum-free medium was served as a control. The neurite outgrowth and influx of calcium in F11 cells among various groups were evaluated. Assay was carried out in duplicate and repeated three times for quantitative analysis.

### Quantitative reverse transcription-polymerase chain reaction

Total RNA was extracted from tissues or cells with the Total RNA Isolation Kit (ES Science, RN001) and reversed by the PrimeScript RT Reagent Kit (TaKaRa) in according with the manufacturer’s recommendations. The SYBR Premix Ex Taq Kit (TaKaRa) was used for PCR, and the fold change was determined through the 2^–△△Ct^ method. The primers were synthesized by Generay Biotechnology, and the sequences are listed in **Additional file 2**.

### Western blot

Samples were homogenized in RIPA buffer (Beyotime Biotechnology) with protease and phosphatase Inhibitor (Thermo Fisher). Western blot analyses were carried out in accordance with standard protocols, and membranes were incubated with anti-FGF2 (1:200, Proteintech), anti-GPR30 (1:100, Abcam), phospho-Mek (1:1000, Cell Signaling Technology), Mek (1:1000, Cell Signaling Technology), phospho-p44/42 MAPK (p-Erk1/2, 1:1000, Cell Signaling Technology), p44/42 MAPK (Erk1/2, 1:1000, Cell Signaling Technology), p-FGFR1 (1:500, Abcam), and GAPDH (1:5000, Proteintech). The membranes were incubated with HRP-conjugated antibodies goat anti-rabbit (1:10000, Abcam) or goat anti-mouse (1:10000, Abcam). They were imaged on the Image Quant LAS 4000 mini biomolecular imager (GE Healthcare Life Sciences) after being applied with EZ-ECL (Biological Industries). The relative protein levels were measured by Image J software (National Institutes of Health) and normalized over the expression of GAPDH.

### Immunohistochemistry and immunofluorescence

Tissues were fixed in 4% paraformaldehyde, embedded in paraffin, and cut into 4 μm sections. The sections were deparaffinized with xylene. For immunohistochemistry, the tissues were stained with anti-FGF2 (1:200, Affinity), and anti-FGFR1 (1:200, Abcam), followed by incubation with corresponding secondary antibody for 60 min. The slides were visualized, adding DAB (3,3′-diaminobenzidine) substrate, counterstained with hematoxylin, and mounted for observation under the microscope. A semi-quantitative evaluation of the immunostaining intensity was carried out using Image J, and the mean optical density (MOD) was used to represent the levels of protein expression. The average MOD of five different fields of a slide was regarded as the expression of the molecule. For immunofluorescence, sections were permeabilized by 0.1% TritonX-100 (Sigma–Aldrich) for 10 min. Unspecific bindings were blocked by using 8% bovine albumin at room temperature for 1 hour. The tissues were incubated overnight at 4°C with anti-tryptase (1:200, Abcam), anti-GPR30 (1:100, Abcam), anti-FGF2 (1:200, Affinity), and anti-PGP9.5 (1:200, Abcam). The next day, the slides were washed three times in PBS and incubated with the secondary antibodies: goat anti-mouse antibody (Alexa Fluor 488, 1:500, Abcam) and donkey anti-rabbit antibody (Alexa Fluor 647, 1:500, Abcam) at room temperature for 1 hour. After staining with 4′,6-diamidino-2-phenylindole, and dihydrochloride (DAPI, Abcam), the tissues were visualized under the confocal microscope (Olympus).

### Intracellular calcium measurements

After the different interventions were conducted, the F11 cells were incubated with Fluo-4 AM reagent (5 μM) in HBSS at 37°C for 30 min. Next, the cells were washed with HBSS three times to remove extracellular Fluo-4 AM. They were incubated with HBSS for another 30 min to allow complete de-esterification of intracellular acetoxymethyl (AM) esters. Calcium influx images were required at 10 s intervals for 400 s with an excitation wavelength of 494 nm by using Olympus FV1200 confocal microscope (Olympus). FGF2 (200 ng/mL, Peprotech) was added 80 s after the start of the recording. Fluo-4 AM fluorescence intensity (FI) within the region of interest in different groups was quantified by NIS-Elements Imaging Software. The relative change in fluorescence *ΔF/F0* was calculated, where *F0* is the baseline fluorescence at the start of the recording. The peak amplitudes of *ΔF* were calculated within 320 s post-stimulus.

### Neurite outgrowth assay

Following different interventions for 24 h, the F11 cells were fixed by 4% paraformaldehyde and permeabilized by 0.1% TritonX-100 (Sigma Aldrich) for 10 min. Unspecific bindings were blocked using 8% bovine albumin at room temperature for 1 hour. Cells were incubated with primary antibody TUBB3 (1:100, Abcam) overnight at 4°C. The next day, the cells were incubated with goat anti-mouse antibody (Alexa Fluor 488, 1:500, Abcam). DAPI was used to visualize cell nucleus. The cells were detected under the Olympus FV1200 confocal microscope (Olympus). The number and length of neurites were calculated by Image J from five separated high-power fields in each group. Each experiment was performed three times.

### ELISA

The concentrations of FGF2 in ascites or cell culture supernatants were quantified by ELISA Kits (Fine Test) in accordance with the manufacturer’s instructions. Concisely, ascites of patients or cell culture supernatants of mast cells were collected, centrifuged at 3000rpm for 10 min to remove cells and debris, and then used for FGF2 concentration measurement. The standard curves were used to determine the concentrations of FGF2 protein level.

### Surgical induction and drug intervention of endometriosis rat model

Six-week-old female non-pregnant Sprague–Dawley rats weighing 200–250 g, were purchased from Shanghai Animal Center, Chinese Academy of Science (Shanghai, CHN) and housed in the barrier facility at a 21°C ± 0.5°C, under 12 h:12 h light-dark cycles. The experiments were carried out in accordance with the National Institutes of Health Guide for the Care and Use of Laboratory Animals. This study was approved by the Ethics Committee of Zhejiang University (No. ZJU20220005). The rats were housed under standard conditions for at least 7 days before the commencement of experiments. They underwent endometriosis surgery as previously described (46). In briefly, after anesthetization, an incision was made on the abdomen of rats. One side of the uterine was removed and cut into four equal parts (around 5 mm^2^). Two pieces of endometrium were sewn at each side of the peritoneum, and the other two were sewn around the mesenteric arteries. In the sham operation group, the abdominal cavity of rat was sutured after the removal of one side of the uterine horn. A sham operation group (n = 6) was used as a blank comparison. The remaining rats were randomly divided into two groups. Vehicle (carboxymethylcellulose sodium 0.5% w/v in saline, CMC-Na) was administered by oral gavage to the control group (n = 6). NSC12, an inhibitor for FGF2/FGFR1 interaction, was dissolved in CMC-Na (n = 6). The NSC12 dosage (5.31 mg/kg/day) was based on previous data from the literature about the effects of NSC12 on other animal models (47). All the drug interventions were started on the week 5 and for the next 2 weeks by oral gavage. The rats were sacrificed at week 7 after endometriosis induction to evaluate the effect of treatments. The rats were anesthetized with isoflurane, and the endometriotic implants, DRGs (T11-L2 segment), and brain were collected. All samples were then processed for biochemical studies.

### Nociceptive testing

Mechanical and thermal paw withdrawal tests were used to measure the hyperalgesia of rats before and every week after surgery (48). In brief, the rats were placed in a cage with a wire mesh floor, and they were free to explore and groom. An electronic von Frey anesthesiometer (Model 2390, IITC/Life Science Instruments, Lowell, CA, USA) with a flexible probe was applied to the sole of the right hind paw. Brisk withdrawal or paw flinching was considered as a positive response. The response force of the von Frey hair that caused the hind paw to withdraw was defined as the mechanical pain threshold (MPT). The paw withdrawal latency for noxious thermal stimuli was determined using an apparatus (Model 33B, IITC/Life Science Instruments, Lowell, CA, USA). The rats were placed in a Plexiglas chamber on a glass plate containing a lightbox. Radiant thermal stimulation was applied by passing a beam of light through a hole in the lightbox aimed at the heel of the right hind paw through the glass sheet. When the rat lifted its foot, the beam was turned off. The time between the start of the measurement beam and the foot lift was defined as the heat source latency (HSL). Each test was repeated five times at 5-min intervals at room temperature (20°C), and the average value from the five measurements was obtained.

### Statistics

Statistical analysis was performed using GraphPad Prism 8.0 (GraphPad Software, USA). A two-tailed Student’s t-test was used to identify statistically significant differences between two groups, and one-way ANOVA was used for comparisons among multiple groups. A nonparametric test was used to compare data variables that do not fit the normal distribution and/or whose variance is not equal among groups. Pearson’s correlation analysis was used to determine the correlations between FGF2 concentration levels and the severity of hyperalgesia. All experiments were performed in triplicate, and all the results were expressed as mean ± standard deviation (SEM). A *p* value of < 0.05 was taken as statistically significant.

## Results

### The number of GPR30-positive mast cells was increased in endometriotic lesions

**Figure 1:**
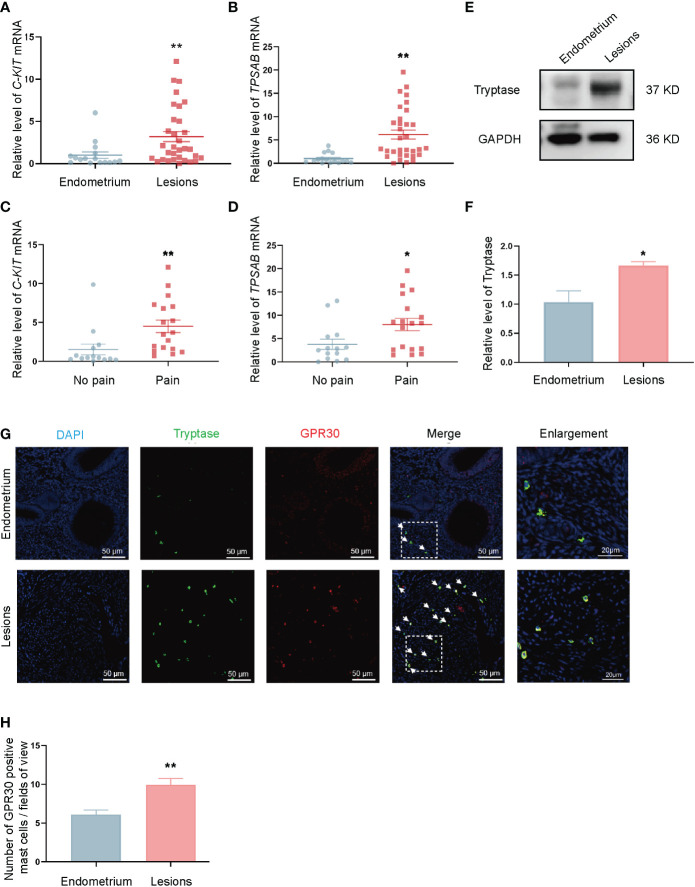
Number of GPR30 positive mast cells is increased in ovarian endometriotic lesions. **(A)** The mRNA level of *C-KIT* in control endometrium (n = 16) and ovarian endometriotic lesions (n = 32). **(B)** The mRNA level of *TPSAB1* in control endometrium (n = 16) and ovarian endometriotic lesions (n = 32). **(C)** The mRNA level of *C-KIT* in ovarian endometriotic lesions from patients with (n = 18) or without (n = 14) endometriosis-related pain. **(D)** The mRNA level of *C-KIT* in ovarian endometriotic lesions from patients with (n = 18) or without (n = 14) endometriosis-related pain. **(E)** Western blot analysis of tryptase in lesions from control endometrium and ovarian endometriotic lesions. **(F)** Relative expression of tryptase in lesions from control endometrium (n = 11) and ovarian endometriotic lesions (n = 16). GAPDH was used as reference protein. **(G)** Representative double immunofluorescence images of tryptase (green) and GPR30 (red) in lesions from control endometrium and ovarian endometriotic lesions; white arrow indicated the positive cells, 400×, bar = 50 μm. Boxed region of the merged images was enlarged on the right, bar = 20 μm. **(H)** Number of GPR30 positive mast cells per fields of view in control endometrium (n = 11) and ovarian endometriotic lesions (n = 16). Endometrium: endometrium tissue from patients without endometriosis, Lesions: ovarian endometriotic lesions from patients. No pain: lesions from patients without endometriosis-related pain, Pain: lesions from patients with endometriosis-related pain. *P < 0.05, **P < 0.01.

RT-qPCR and Western-blot were conducted to detect mast cells in endometriotic lesions and control endometrium tissue. *C-KIT* (*KIT* proto-oncogene, receptor tyrosine kinase) is a gene that encodes a mast-cell surface marker named Kit receptor (alias CD117). *TPSAB1* (tryptase alpha/beta 1) is a gene that encodes a mast-cell-specific proteases in mast cell granules called tryptase. The mRNA levels of mast-cell-specific markers *C-KIT* and *TPSAB1* in ovarian endometriotic lesions were found to be increased compared with those in control endometrium tissue (**Figures 1A, B**). Moreover, the mRNA levels of *C-KIT* and *TPSAB1* were higher in patients with endometriosis-related pain than those without it (**Figures 1C, D**). An up-regulated level of tryptase protein was also observed in the ovarian endometriotic lesions compared with control endometrium (**Figures 1E, F**). Double immunofluorescence staining was applied in the ovarian endometriotic lesions of patients and control endometrium tissues from patients without endometriosis to further investigate the expression of GPR30 in mast cells distributed around ovarian endometriotic tissues (**Figure 1G**). The results indicated that the number of GPR30-positive mast cells per fields of view were significantly higher in the ovarian endometriosis lesion group than in the control endometrium groups (**Figure 1H**).

### Neurite growth and Ca^2+^ influx in DRG cells was promoted by mast cells *via* non-classic estrogen receptor

**Figure 2:**
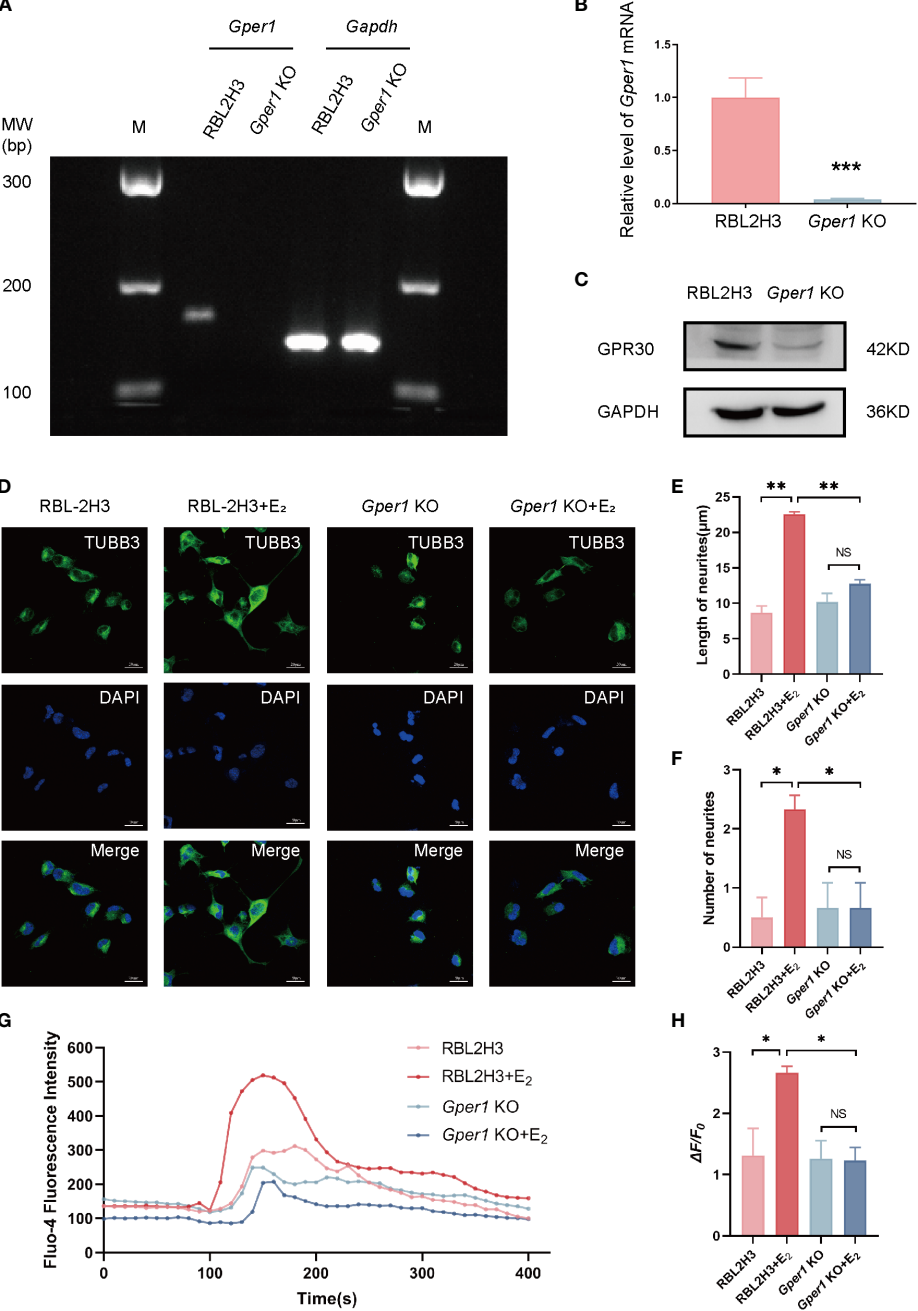
Mast cells promote the neurite growth and Ca2+ influx in DRG cells *via* GPR30. **(A)** Expression of *Gper1* gene in mast cells, as examined by agarose gel electrophoresis (n = 3 in each group). **(B)** *Gper1* expression in mast cells, as examined by RT-qPCR (n = 6 in each group). **(C)** GPR30 protein expression in mast cells, as examined by western blot (n = 3 in each group). **(D)** Representative images of cell morphology and neurite growth in DRG cells treated with different supernatants from mast cells for 24 h. **(E)** Quantified results of length neurites in different groups (n = 4, 5, 3, 3). **(F)** Quantified results of number of neurites in different groups (n = 6, 9, 6, 6). **(G)** Fluo-4 fluorescence intensity of F11 cells treated with different supernatants from mast cells. **(H)** Ca^2+^ peak increase (*ΔF/F0*) in F11 cells treated with different supernatants from mast cells (n = 3 in each group). The concentration of E_2_ is 100 nM. *P < 0.05, **P < 0.01, NS, not significant.

A CRISPR-Cas9 gene editing system was generated to introduce GPR30 (*Gper1*) knockout cell lines to explore the basic function of GPR30 in mast cells. Establishment of *Gper1* knockout cells was confirmed by PCR (**Figure 2A**), RT-qPCR (**Figure 2B**), and Western blot (**Figure 2C**). The results indicated that the expression levels of *Gper1* and GPR30 were lower in the *Gper1* KO RBL2H3 cells than in the RBL2H3 cells. RBL2H3 cells and *Gper1* KO RBL2H3 cells were treated with or without 100 nmol/L of 17β-estradiol to assess the function of non-classical estrogen pathway in mast cells. After 24 h, the supernatants of the four groups were collected and added to DRG cells (F11 cells) instead of culture medium. The length and the number of neurites were evaluated 24 h later. The number and length of the neurites in the F11 cells of the RBL2H3 + E_2_ group notably increased compared with those of the RBL2H3 supernatants group. However, this phenomenon was not detected in the *Gper1* KO RBL2H3 cells. (*P*<0.05; **Figures 2D–F**). Calcium influx was carried out to evaluate the neuro-sensitization of DRG cells. The intracellular Ca^2+^ level of F11 cells was evaluated by Fluo-4 AM after treating with supernatants from the four groups. An increase in the Fluo-4 fluorescence intensity in the group of RBL2H3 cells treated with 100 nmol/L of 17β-estradiol was observed, indicating the supernatants of the RBL2H3 cells treated with 100 nmol/L E_2_ promoted the Ca^2+^ influx of F11 cells (**Figure 2G**). The mean amplitude of calcium peaks (*ΔF/F0*) in the RBL2H3 + E_2_ group were enhanced compared with that in the RBL2H3 group (*P* < 0.05; **Figure 2H**). No significant difference was found between the *Gper1* KO and *Gper1* KO + E_2_ groups (*P* > 0.05; **Figure 2H**). Taken together, these results showed that GPR30 could mediate the activation of non-classic estrogen pathway in mast cells. In addition, the supernatants of mast cells after pathway activation could promote the neurite growth and Ca^2+^ influx in DRG cells. The phenomenon above provides important evidence of mast-cell-neuron direct communication. The underlying mechanism was subsequently explored.

### 17β-estradiol upregulated the FGF2 by activating the GPR30/p-MEK/p-ERK signaling pathway in mast cells

**Figure 3:**
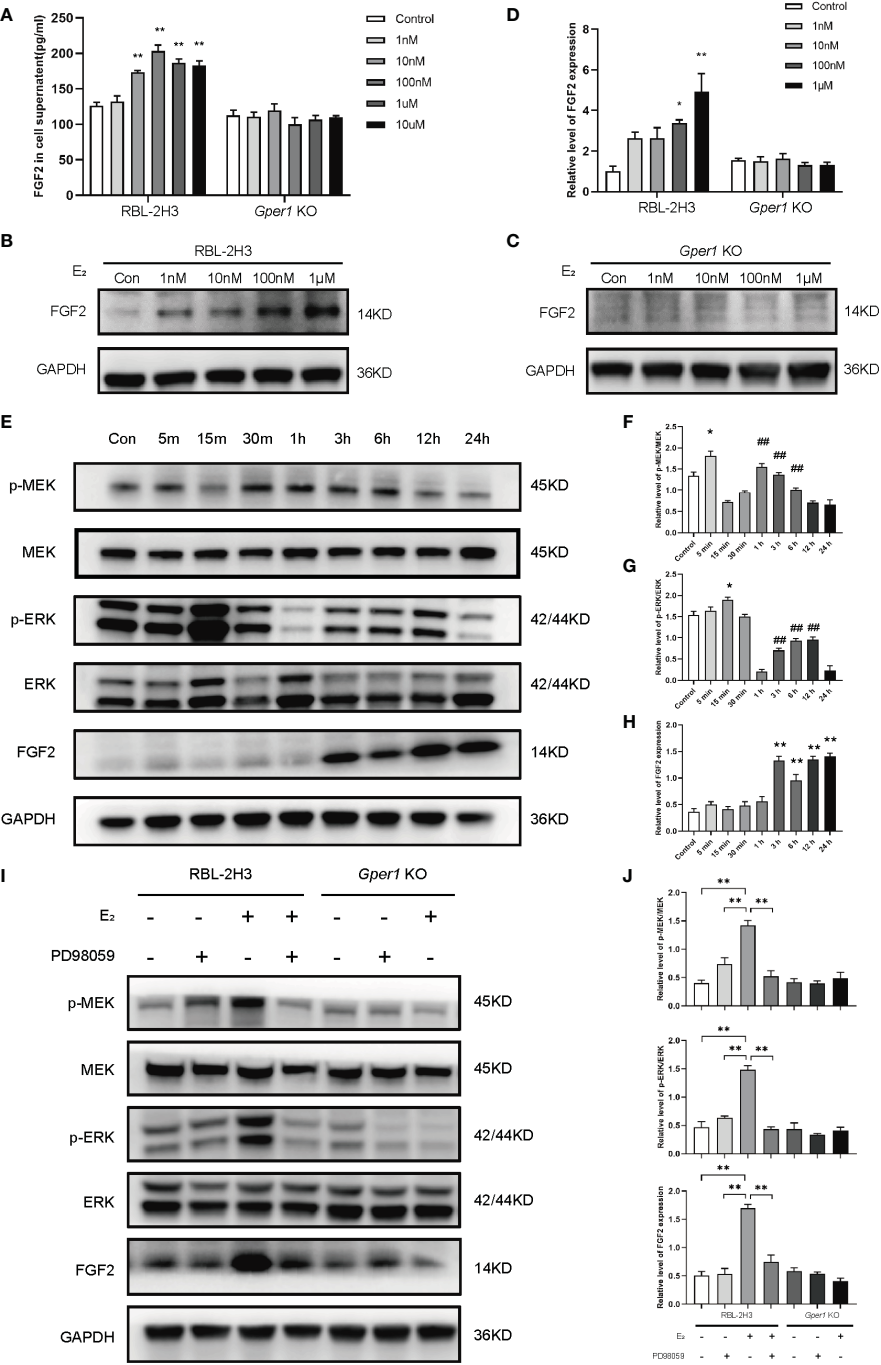
Upregulated FGF2 in mast cells depends on the activation of GPR30/p-MEK/p-ERK signaling pathway. **(A)** Contents of FGF2 in the supernatant of mast cells after intervention with different concentrations of estrogen. **(B)** Protein expression of FGF2 in RBL2H3 cells after intervention with different concentrations of estrogen. **(C)** Protein expression of FGF2 in *Gper1* KO RBL2H3 cells after intervention with different concentrations of estrogen. **(D)** Relative expression of FGF2 protein in two mast cells after intervention with different concentrations of estrogen. **(E)** Western blot analysis of p-MEK, MEK, p-ERK, ERK, and FGF2 in mast cells after estrogen (100 nM) treatment at various timepoints. **(F)** Relative level of p-MEK/MEK in mast cells after estrogen (100 nM) treatment at various timepoints. * P < 0.05 (5 min vs. Control), ## P < 0.01 (1, 3, 6 h vs. 15 min).**(G)** Relative level of p-ERK/ERK in mast cells after estrogen (100 nM) treatment at various timepoints. * P < 0.05 (15 min vs. Control), ## P < 0.01 (1, 3, 6, 12 h vs. 1 h).**(H)** Relative expression of FGF2 in mast cells after estrogen (100 nM) treatment at various timepoints. ** P < 0.05 (3, 6, 12, 24 h vs. Control).

The concentration of FGF2 in the supernatant of mast cells were evaluated by ELISA. The FGF2 levels in RBL2H3 cells increased when treated with 100 nmol/L, 1 μmol/L, 10 μmol/L of 17β-estradiol compared with those in the control group (*P* < 0.01; **Figure 3A**). However, no alternation was found in the supernatants of *Gper1* KO cells after various concentrations of E_2_ intervention (*P* > 0.05; **Figure 3A**). The results of Western blot were consistent with the protein level detected in the supernatants. **Figure 3B** shows that the protein level of FGF2 was increased in RBL2H3 cells increased after treatment with 1 nmol/L, 10 nmol/L, 100 nmol/L, 1 μmol/L of 17β-estradiol (*P* < 0.05; **Figures 3B, D**), whereas no alternation was found in the protein level in the *Gper1* KO RBL2H3 groups after 17β-estradiol treatment (**Figures 3C, D**). The level of phospho-proteins and FGF2 in RBL2H3 cells and *Gper1* KO RBL2H3 cells were evaluated after treatment with 17β-estradiol (100 nM) at different time points (5, 15, and 30 min, and 1, 3, 6, 12 and 24 h) to explore the related signaling pathway. The results of Western blot indicated that the protein levels of p-MEK and p-ERK in RBL2H3 cells significantly increased after 17β-estradiol intervention when comparing with those without 17β-estradiol treatment (**Figures 3E–H**). Moreover, p-MEK expression was significantly increased at 30 min and showed a decreased trend (**Figures 3E, F**). The peak expression levels of p-ERK were at 15 min and 3 h (**Figures 3E, G**). The protein level of FGF2 was increased significantly after 3 h. For confirmation of the above results, the MEK inhibitor PD98059 was introduced. PD98059 (10 μM) preincubation for 1 h significantly reduced the phosphorylation of p-MEK and p-ERK and up-regulated the production of FGF2. Overall, these results indicated that the enhanced secretion of FGF2 in mast cells was induced by 17β-estradiol through the GPR30/p-MEK/p-ERK signaling pathway.

### Increased expression of FGF2 by mast cells around the nerve structures in endometriotic lesion was associated with pain

**Figure 4:**
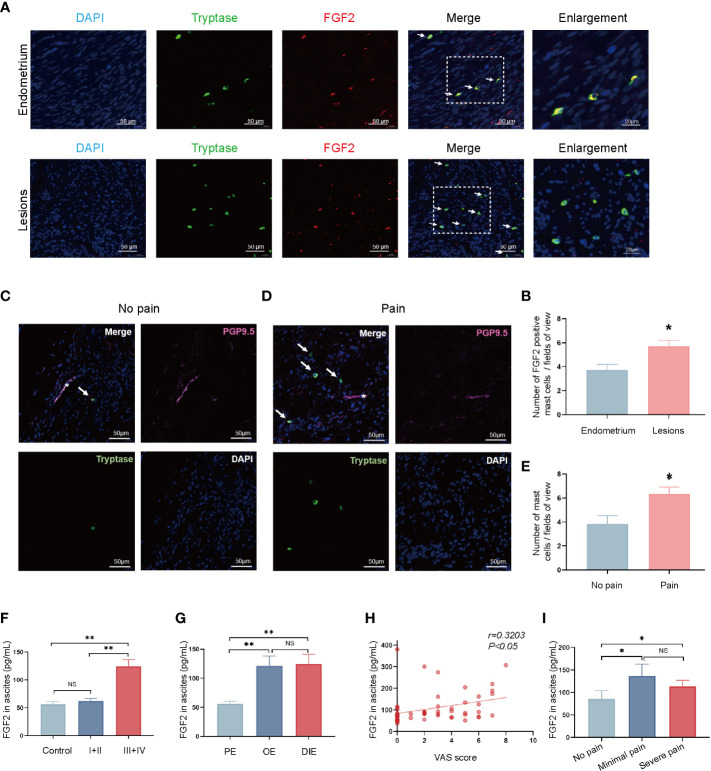
Pain severity of patients is related to increase in FGF2 around the nerve structures in endometriotic lesion. **(A)** Representative double immunofluorescence images of tryptase (green) and FGF2 (red) in lesions from control endometrium and ovarian endometriotic lesions; white arrow indicated the positive cells, 400×, bar = 50 μm. Boxed region of the merged images was enlarged on the right, bar = 20 μm. **(B)** Quantitative numbers of FGF2 positive mast cells per field of view in control endometrium (n = 11) and ovarian endometriotic lesions (n = 16). **(C)** Representative double immunofluorescence images of tryptase (green) and PGP9.5 (rose red) in lesions from patients without endometriosis-related pain; white arrow indicated the positive cells, 400×, bar = 50 μm. **(D)** Representative double immunofluorescence images of tryptase (green) and PGP9.5 (rose red) in lesions from patients with endometriosis-related pain symptoms; white arrow indicated positive cells, 400×, bar = 50 μm. **(E)** Quantitative numbers of tryptase positive mast cells per field of view in control endometrium (n = 7) and endometriotic lesions (n = 9). **(F)** Concentration of FGF2 in the peritoneal fluid of control patients without endometriosis and patients with endometriosis in different stages (n = 16, 14, 39). **(G)** Concentration of FGF2 in the peritoneal fluid of different types of endometriosis, PE, peritoneal endometriosis; OE, ovarian endometrioma; DIE, deep infiltrated endometriosis (n = 12, 24, 17). **(H)** Correlation between FGF2 level in the peritoneal fluid and visual analogue scale (VAS) score of patients (n = 53, r = 0.1026, *P*<0.05). **(I)** Concentration of FGF2 in the peritoneal fluid of endometriosis patients with different pain symptoms (n = 18, 8, 27). *P < 0.05, **P < 0.01, NS, not significant.

This study’s focus moved on to women with endometriosis to clarify the role of mast-cells-derived FGF2 in endometriosis-related pain. Immunofluorescence staining showed co-expression of tryptase (green) and FGF2 (red) in ovarian endometriotic tissue (**Figure 4A**), which indicated that FGF2 was derived from mast cells. The number of FGF2 positive mast cells were increased in ovarian endometriosis lesions than control endometrium from non-endometriosis patients (**Figure 4B**). The role of mast cells in chronic pain has long been discussed. For determination of whether the mast cells around the nerve structures contribute to the symptoms of endometriosis-related pain, double immunofluorescence was performed in paraffin sections of ovarian endometriotic lesions in women with or without pain. The mast cells were marked by tryptase (green), and the nerve structures were labeled by PGP9.5 (rose red). The sections of ovarian endometriosis lesions were divided into two groups according to patients’ pain symptom. As shown in **Figures 4C, D**, the sections from the pain group contained more tryptase-positive mast cells located within the nerve structures than the sections from the no-pain group. The number of tryptase-positive mast cells was correlated with pain symptoms (**Figure 4E**). These results indicated that the accumulation of mast cells around the nerve structures may contribute to endometriosis-related pain. A significant increase in the concentration of FGF2 was observed in the peritoneal fluid of patients with endometriosis in stages III and IV compared with the control patients and those in stages I and II (**Figure 4F**). The FGF2 level was higher in patients with ovarian endometriotic cyst and deep infiltrated endometriosis lesions than in patients with peritoneal endometriotic lesions (**Figure 4G**). In addition, the Visual Analogue Scale (VAS) score of patients with dysmenorrhea was correlated with FGF2 level in pelvic fluid (r = 0.1026, *P* < 0.05; **Figure 4H**). Moreover, the FGF2 concentration in pelvic fluid was higher in the minimal-pain group and severe-pain group than in patients without endometriosis-related pain (**Figure 4I**). Taken together, these results suggested that the enhanced secretion of FGF2 in mast cells may be a potential cause of endometriosis-associated pain in women.

### Increased FGFR1 expression in endometriotic lesions was correlated with pain symptom of patients with endometriosis

**Figure 5:**
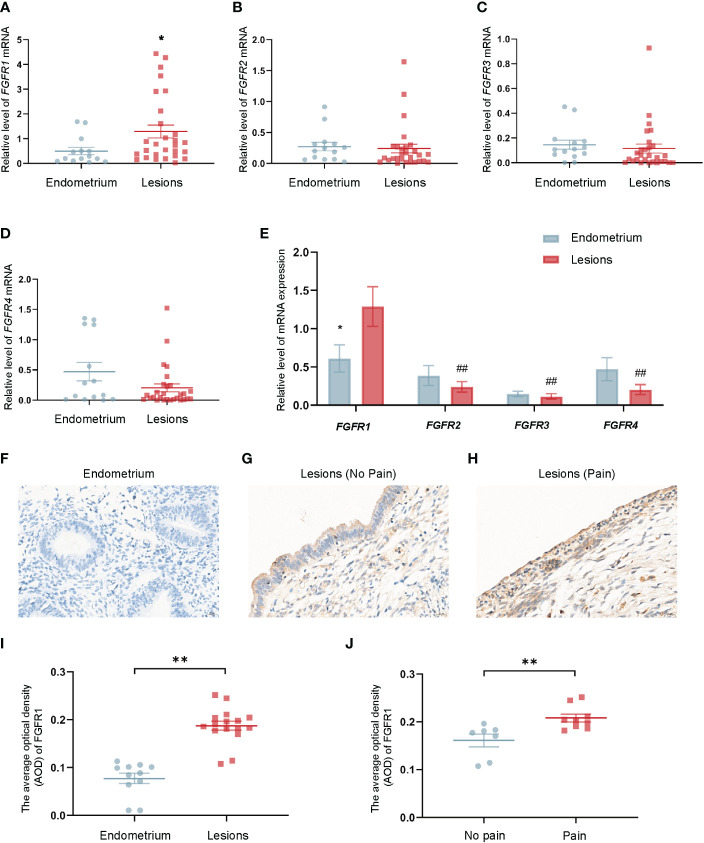
Upregulated FGFR1 in ovarian endometriotic lesions is correlated with patients’ pain severity. **(A)** The mRNA level of *FGFR1* in control endometrium (n = 14) and ovarian endometriotic lesions (n = 28). **(B)** The mRNA level of *FGFR2* in control endometrium (n = 14) and ovarian endometriotic lesions (n = 28). **(C)** The mRNA level of *FGFR3* in control endometrium (n = 14) and ovarian endometriotic lesions (n = 28). **(D)** The mRNA level of *FGFR4* in control endometrium (n = 14) and ovarian endometriotic lesions (n = 28). **(E)** The mRNA level of *FGFR1,2,3,4* in control endometrium (n = 14) and ovarian endometriotic lesions (n = 28). **(F)** Representative IHC images of FGFR1 in tissues from control endometrium, 400×, bar = 20 μm. **(G)** Representative IHC images of FGFR1 in ovarian endometriotic lesions from patients without pain, 400×, bar = 20 μm. **(H)** The representative IHC images of FGFR1 in ovarian endometriotic lesions from patients with pain symptoms, 400×, bar = 20 μm. **(I)** Average optical density of FGFR1 in control endometrium (n = 11) and ovarian endometriotic lesions (n = 16). **J** Average optical density of FGFR1 in ovarian endometriotic lesions from patients without (n = 7) and with (n = 9) pain symptoms. *P < 0.05, **P < 0.01 (control endometrium vs. ovarian endometriosis lesions; no pain vs. pain); ^##^ *P* < 0.01 (*FGFR2*, *FGFR3*, *FGFR4* vs. *FGFR1*).

Various of FGFR1 in human tissues were investigated using qRT-PCR to understand the mechanism of FGF2 function. The results showed the mRNA level of *FGFR1* in ovarian endometriotic lesions in patients with endometriotic were higher than that in control endometrium tissues (* *P* < 0.05; **Figures 5A–D**). Notably, the mRNA level of FGFR1 was the highest in patients with endometriosis among these receptors (^##^ *P* < 0.05; **Figure 5E**). Moreover, IHC staining of FGFR1 was applied in control endometrium tissues and ovarian endometriotic lesions. The expression level of FGFR1 was found to be upregulated in the ovarian endometriotic lesions group (**Figures 5F, G, I**). Ovarian endometriotic tissues from endometriosis patients with endometriosis-related pain symptoms had a higher FGFR1 level than those from patients without endometriosis-related pain (**Figures 5G, H, J**).

### FGF2 promoted the neurite growth and Ca^2+^ influx in DRG cells *via* fibroblast growth factor receptor 1

**Figure 6:**
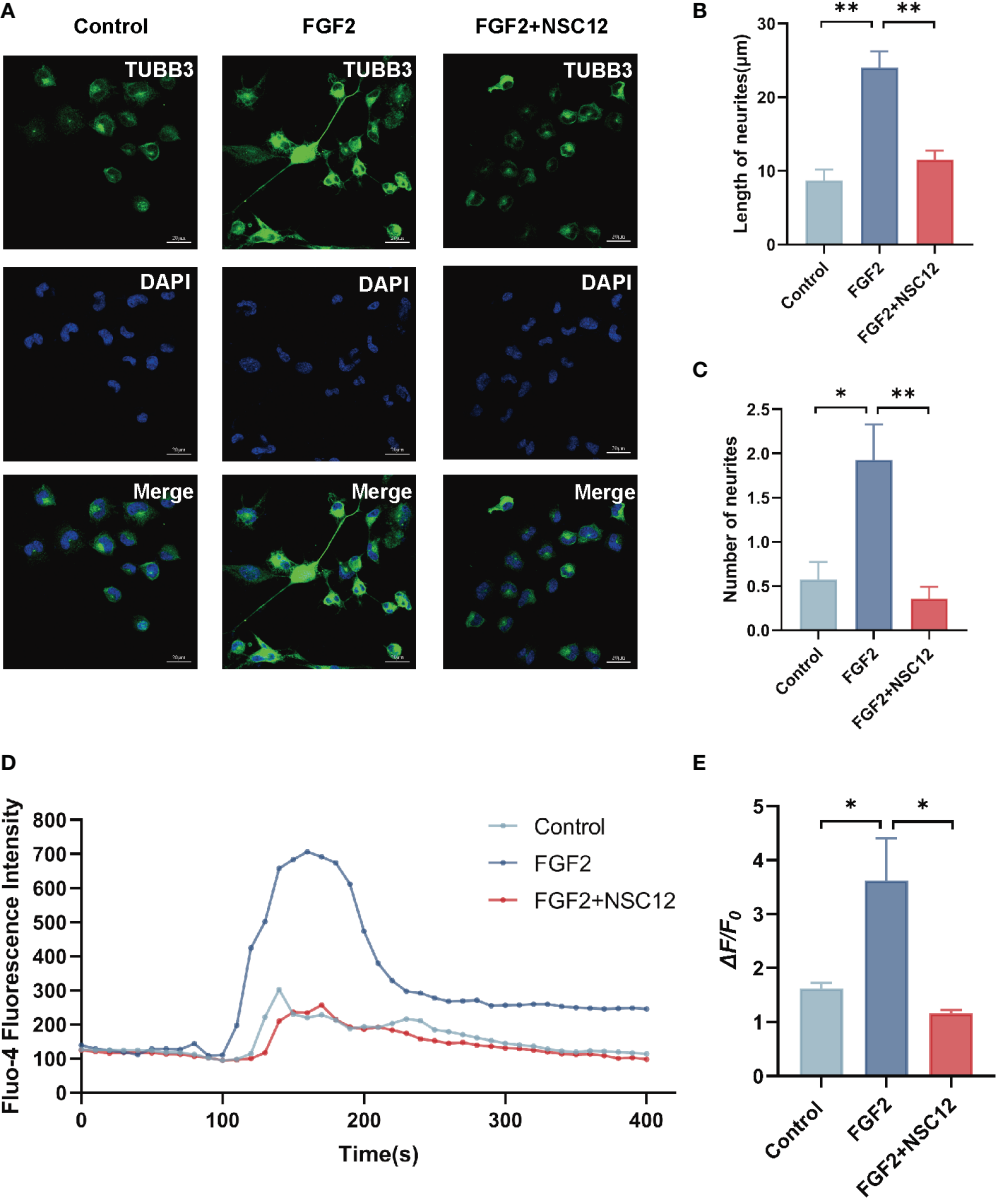
NSC12 could reverse the function of FGF2 in promoting neurite growth and Ca^2+^ influx in DRG cells. **(A)** Representative images of cell morphology and neurite growth in DRG cells treated by FGF2 (200 ng/mL) with or without preincubation of NSC12 (10 μmol/L). **(B)** Quantified results of length of neurites in different groups (n = 4, 5, 3). **(C)** Quantified results of number of neurites in different groups (n = 14, 14, 14). **(D)** Fluo-4 fluorescence intensity of F11 cells treated by FGF2 with or without incubation of NSC12. **(E)** Ca^2+^ peak increase (*ΔF/F0*) in F11 cells treated by FGF2 with or without incubation of NSC12 (n = 3 in each group). *P < 0.05, **P < 0.01.

In the subsequent experiment, NSC12, a FGF2/FGFR1 specially inhibitor, was selected to explore the mechanism of FGF2 in F11 cells. As shown in the results, FGF2 significantly promoted the length and number of neurites in F11 cells compared with those in the control group (*P* < 0.001 and *P* < 0.05, respectively; **Figures 6A–C**). The effects of FGF2 in promoting the length and number of neurites in F11 cells were blocked by NSC12 incubation (*P* < 0.01; **Figures 6A–C**). The Fluo-4 fluorescence intensity and the mean amplitude of calcium peaks (*ΔF/F0*) of F11 cells in the FGF2 treatment group increased (*P* < 0.05; **Figures 6D, E**). Meanwhile, NSC12 pre-incubation decreased the Fluo-4 fluorescence intensity and the mean amplitude of calcium peaks (*ΔF/F0*) in F11 cells (*P* < 0.05; **Figures 6D, E**). The results above showed that NSC12 could reverse the effect of FGF2 in promoting the neurite growth and Ca^2+^ influx in F11 cells.

### NSC12 attenuated endometriosis-induced hyperalgesia in rat models

**Figure 7:**
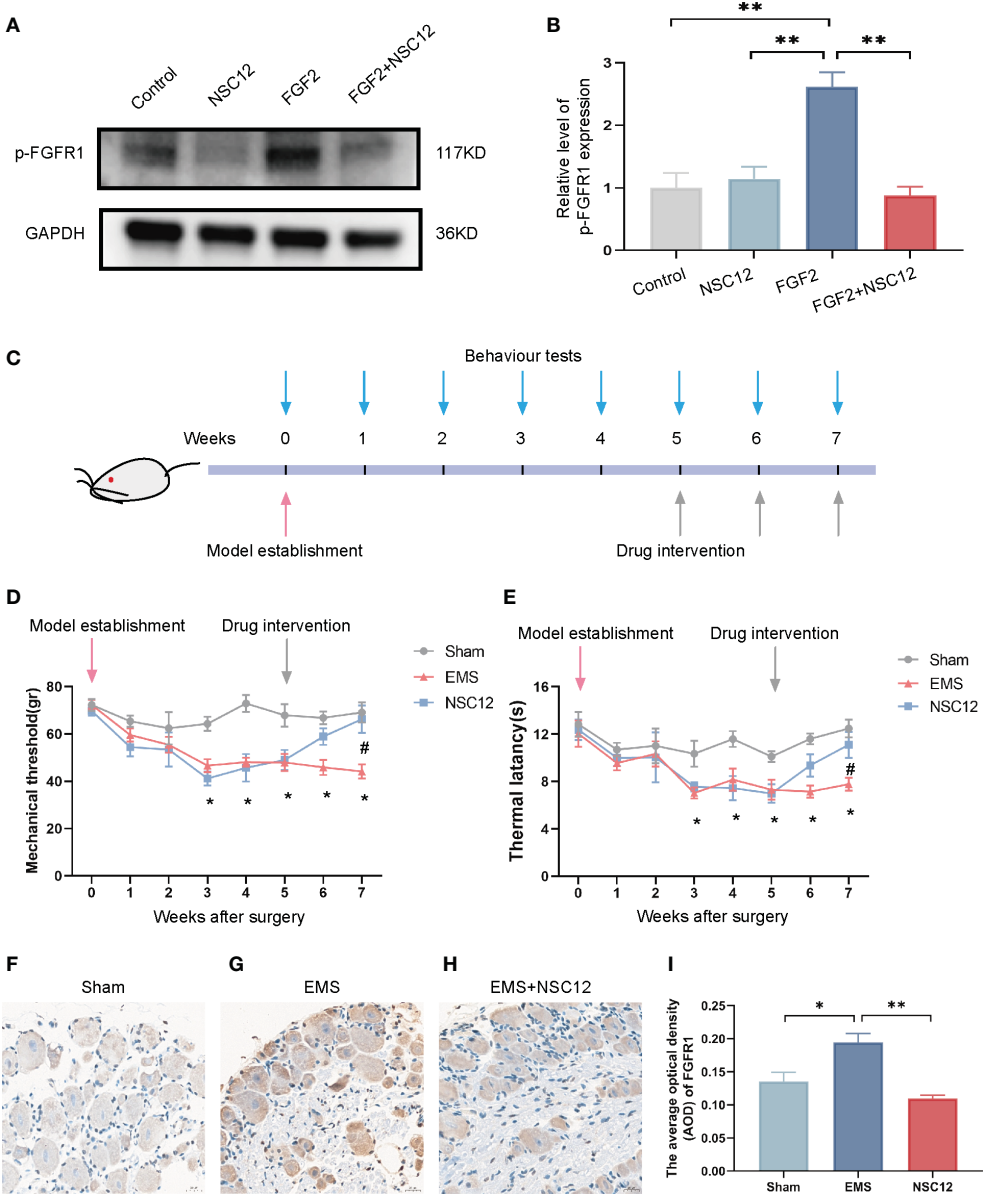
NSC12 relieves endometriosis-induced hyperalgesia *in vivo*. **(A)** Protein expression of p-FGFR1in DRG cells after different interventions. **(B)** Relative level of p-FGFR1 expression (n = 3 in each group). **(C)** Schematic of animal intervention. **(D)** Mechanical threshold after different interventions (n = 6 in each group). **(E)** Thermal latency after different interventions (n = 6 in each group). **(F–H)** The Representative images of p-FGFR1 staining in DRG tissue, 400×, bar = 20 μm. I The Relative expression of p-FGFR1 in DRG tissues different intervention group (n = 6, 6, 5). Sham: sham operation group, EMS, endometriosis model; NSC12, endometriosis model establishment and NSC12 intervention. *P < 0.05, **P < 0.01.

The efficacy of NSC12 in relieving endometriosis-induced hyperalgesia was explored in depth. In **Figures 7A, B**, the protein level of p-FGFR1 was up-regulated after FGF2 treatment in DRG cells, whereas NSC12 administration reversed the increased level of protein expression. Pain-related behavioral changes could be found in endometriosis rats 3 weeks after surgical intervention, and they lasted until week 7 after surgery (**Figure 7C**). A clear trend of decreasing of mechanical threshold and thermal latency could be observed in the endometriosis rats when compared with the sham operation group (*P < 0.05; **Figures 7D, E**). The mechanical threshold and thermal latency were increased after NSC12 intervention for 2 weeks compared with the endometriosis rats (*P < 0.05; **Figures 7D, E**). The IHC staining indicated the protein level of p-FGFR1 in DRG tissues from endometriosis rats were increased, whereas the p-FGFR1 level downregulated after NSC12 administration (*P < 0.05; **Figures 7F–I**). These findings showed that NSC12 could increase the mechanical threshold and thermal latency, thus attenuating endometriosis-induced hyperalgesia in rat models.

## Discussion

**Figure 8:**
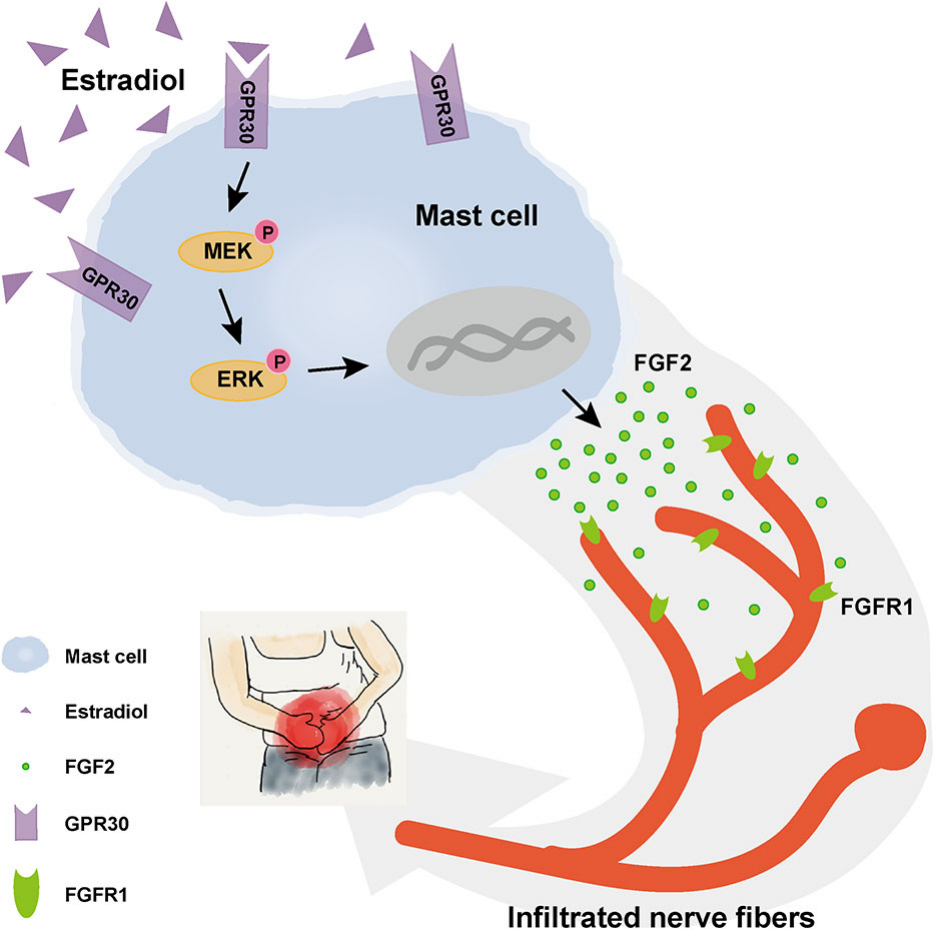
Schematic diagram of the present findings. High concentration of estrogen in local endometriotic environment could promote the secretion of FGF2 through GPR30 *via* MEK/ERK pathway in mast cells. Enhanced level of FGF2 by mast cells in ovarian endometriotic lesion may aggravated endometriosis-related pain.

This research revealed the activation of GPR30-mediated non-classic estrogen pathway in mast cells in endometriotic lesions with high local estrogen, and the potential mechanism of mast-cells-derived FGF2 in endometriosis-related pain was explored, as shown in **Figure 8**.

Former studies have reported that mast cells were detected in normal endometrium (49), eutopic endometrium and different types of lesions from endometriosis patients (19, 50). Estrogen could trigger the release of various mediators in mast cells *via* estrogen receptors expressed on mast cells (14, 51). Previous studies have found that the expression level of GPR30 in endometriotic lesions and eutopic endometrium is higher than that in control endometrium (52, 53). However, the downstream pathway followed by GPR30 activation and the function of mast-cell-derived mediators in endometriosis-related pain remain uncertain. Here, the estrogen-mediated secretion of FGF2 from mast cells *via* GPR30 was found to be dependent on the activation of the MEK/ERK pathway. This effect was suppressed by the MEK inhibitor PD98059. Mast cells tend to recruit around endometriotic lesions, thus providing a microenvironment that allows the formation and progression of endometriotic lesions and endometriosis-related pain (54–56). At present, the drugs that targeting mast cells in the human body are mainly divided into mast cell stabilizers (such as sodium cromolate, nedocromil and lodoxamide, etc.), those with dual effects of mast cell stabilizers and histamine H1 receptor antagonists antiallergic drugs (such as ketotifen, olopatadine and azelastine) (57), and those targeting mast-cell degranulation, such as ketotifen (58), Janus kinase 3 inhibitors (59), phosphoinositide 3 kinase, and pathway neurokinin receptor antagonism (60). However, the preclinical and clinical trials of drugs with mast cells as potential therapeutic targets in endometriosis and endometriosis-related pain still lack sufficient attention and deep research.

According to literature reports, related studies on mast-cell-derived FGF2 mainly focus on angiogenesis, fibrosis, wound healing, and diseases involving hypertensive renal damage, airway hyperresponsiveness, chronic obstructive pulmonary disease and other diseases (35, 37, 38, 61). Several studies have reported the elevated FGF2 in lesions (39, 40), peritoneal fluid (41), or peripheral blood in patients with endometriosis (42, 43). The role and function mechanism of FGF2 in endometriosis-related pain deserves in-depth study. In previous studies, the receptors which FGF2 binds to are often one of the important questions that researchers attempt try to explain. Christine et al. found that FGFR1 was expressed in DRG neurons of the lumbar spine of rats, and intradermal injection of FGF2 in the plantar of rats could induce mechanical pain sensitivity in rats (62). Zhang et al. found that the level of p-FGFR1 was increased in patients with myofascial pain syndrome. In a rat model of myofascial pain syndrome, the protein expression levels of FGF2 and p-FGFR1 were increased and the mechanical pain threshold was decreased (63), suggesting a role for FGF2/FGFR1 in myofascial pain. The results of the present work showed that increased concentration of FGF2 around nerve fibers in endometriosis interact with elevated FGFR1 level on nerve fibers and trigger endometriosis-related pain symptoms.

FGFR1 belongs to the receptor tyrosine kinase family, which plays a role by activating downstream signaling after binding to ligands (64). FGFR1 inhibitors are used in the treatment of various diseases. Zhang et al. reported that the FGFR1 inhibitor PD173074 effectively inhibited p-FGFR1 phosphorylation and increased the threshold for mechanical pain in rats with myofascial pain syndrome (65). NSC12 is a small molecule inhibitor that could orally inhibit the interaction of FGF2/FGFR. Studies have shown that it has a high-efficiency FGF2/FGFR inhibitory effect in lung cancer, melanoma, and multiple myeloma (66, 67). The present study showed that NSC12 could inhibit FGF2-induced calcium influx and axonal growth in DRG cells at the cellular level. NSC12 proved efficacious in the relief of pain sensitization in rats, suggesting that NSC12 may act as a novel modality for the treatment of endometriosis-related pain. An in-vivo experiment found that AZD4547 could attenuated endometriotic lesion formation without affecting estrous cycle (68). Deep investigation is needed to clarify the effect of NSC12 in estrous cycle before further trial. Inhibition of FGFR1 by shFGFR1 in an endometriosis model of nude mice could prevent lesion formation and dysmenorrhea severity (69), also providing a new direction in targeting FGFR1 in endometriosis.

The advantage of this study is that we constructed the *Gper1* KO mast cells were generated by CRISPR/Cas9 to establish an experimental basis for the subsequent verification of the function of GPR30. However, this study still has some limitations. Firstly, the human sample of human is insufficient, and needing expansion. Secondly, the eutopic endometrium group from patients with endometriosis was not involved. Most patients with endometriosis have reproductive needs, and the collection of eutopic endometrial samples during surgery may affect the function of endometrium, so we did not set the eutopic endometrium group. Thirdly, the mast cell and DRG cell line may not be representative enough, and a better model, such as primary culture cells, need to be explored.

Collectively, by using a combination of in-vivo and -vitro models, we found enhanced secretion of FGF2 in mast cells was found to be induced by estrogen *via* a non-classic signaling pathway. The increased FGF2 plays a vital role in the mast cell-nerve crosstalk, which contributes to endometriosis-related pain. Targeting this process may be a promising strategy for relieving endometriosis-related pain.

## Data availability statement

The original contributions presented in the study are included in the article/**Supplementary Material**. Further inquiries can be directed to the corresponding author.

## Ethics statement

The studies involving human participants were reviewed and approved by The Human Ethics Committee of Women’s Hospital, School of Medicine, Zhejiang University (No. 20160114). The patients/participants provided their written informed consent to participate in this study. The animal study was reviewed and approved by The Committee on the Ethics of Animal Experiments of Zhejiang University (No. ZJU20220005). Written informed consent was obtained from the individual(s) for the publication of any potentially identifiable images or data included in this article.

## Author contributions

XX wrote the manuscript. XX and JW designed the experiments. XG, XX, TL, YC and SD performed the experiments and analyzed the data. GZ, LZ and TL compiled the figures. JW, XZ and SD reviewed the manuscript. All authors contributed to the article and approved the submitted version.
