# Supplementary material for: GPR30-mediated non-classic estrogen pathway in mast cells participates in endometriosis pain via the production of FGF2
Source: Front Immunol. 2023 Feb 8;14:1106771. doi: 10.3389/fimmu.2023.1106771 (PMC9945179; doi:10.3389/fimmu.2023.1106771)
Supplement: Supplementary file 1 [file Table_1.pdf]

Table.1 Patient information of tissue sample

	Endometriosis（n=32）	Non endometriosis（n=16）	*P*
Age（year）	33.1±1.0	34.3±1.7	0.519
Pregnant	1.0±0.2	1.3±0.4	0.382
Labor	0.5±0.1	0.7±0.2	0.296
Abortion	0.5±0.1	0.6±0.3	0.565
Menstrual cycle			
Proliferative phase	21（68.6%）	13（81.2%）	
Secretory phase	11（34.4%）	3（18.8%）	
Pain symptom			
No Pain	14（43.8%）		
Pain	18（56.2%）		

Table. 2 Patient information of peritoneal fluid sample

	Endometriosis（n=53）	Non endometriosis（n=16）	*P*
Age（year）	33.9±1.1	34.0±2.9	0.964
Pregnant	1.2±0.2	1.5±0.3	0.401
Labor	0.6±0.1	0.6±0.1	0.990
Abortion	0.5±0.1	0.9±0.2	0.217
Menstrual cycle			
Proliferative phase	36（67.9%）	9（56.3%）	
Secretory phase	17（32.1%）	6（37.5%）	
rAFS stage			
I	12（22.6%）		
II	2（3.8%）		

	Endometriosis（n=53）	Non endometriosis（n=16）	*P*
III	14（26.4%）		
IV	25（47.2%）		
Type			
Peritoneal lesion	12（22.6%）		
Ovarian endometrioma	24（45.3%）		
DIE	17（32.1%）		
Pain symptom			
No Pain	20（37.7%）	12（75.0%）	
Pain	33（62.3%）	4（25.0%）	

rAFS: revised American Fertility Society; DIE: deep infiltrated endometriosis.
