# Supplementary material for: GPR30-mediated non-classic estrogen pathway in mast cells participates in endometriosis pain via the production of FGF2
Source: Front Immunol. 2023 Feb 8;14:1106771. doi: 10.3389/fimmu.2023.1106771 (PMC9945179; doi:10.3389/fimmu.2023.1106771)
Supplement: Supplementary file 2 [file Table_2.pdf]

Primer sequences of quantitative real-time PCR

**Primer**	**Forward (5'-3')**	**Reverse (5'-3')**
GAPDH(Human)	GGAGCGAGATCCCTCCAAAAT	GGCTGTTGTCATACTTCTCATGG
C-KIT(Human)	GCGTTCTGCTCCTACTGCTTCG	TGGATGGATGGTGGAGACGGTTC
TPSAB1(Human)	GTGACGCAAAATACCACCTTGGC	CCATTCACCTTGCACACCAGGG
FGFR1(Human)	CCCGTAGCTCCATATTGGACA	TTTGCCATTTTTCAACCAGCG
FGFR2(Human)	GGTGGCTGAAAAACGGGAAG	AGATGGGACCACACTTTCCATA
FGFR3(Human)	TGCGTCGTGGAGAACAAGTTT	GCACGGTAACGTAGGGTGTG
FGFR4(Human)	CCATAGGGACCCCTCGAATAG	CAGCGGAACTTGACGGTGT
GAPDH(Rat)	CTCATGACCACAGTCCATGC	TTCAGCTCTGGGATGACCTT
GPR30(Rat)	AGTTTCGGCGCTGATGGTT	GGTCTTCTTCCTCTGCGGGTA
